# A Novel RNA Synthesis Inhibitor, STK160830, Has Negligible DNA-Intercalating Activity for Triggering A p53 Response, and Can Inhibit p53-Dependent Apoptosis

**DOI:** 10.3390/life11101087

**Published:** 2021-10-15

**Authors:** Akinori Morita, Shintaro Ochi, Hidetoshi Satoh, Shohei Ujita, Yosuke Matsushita, Kasumi Tada, Mihiro Toyoda, Yuichi Nishiyama, Kosuke Mizuno, Yuichi Deguchi, Keiji Suzuki, Yoshimasa Tanaka, Hiroshi Ueda, Toshiya Inaba, Yoshio Hosoi, Shin Aoki

**Affiliations:** 1 Tokushima University, Tokushima 770-8503, Japan; ochis@opho.jp (S.O.); shoheiujita@gmail.com (S.U.); y-matsushita@genome.tokushima-u.ac.jp (Y.M.); kasumitada15@gmail.com (K.T.); mifusagimif@gmail.com (M.T.); y-nishi@tokushima-u.ac.jp (Y.N.); 2 Department of Medicinal and Life Science, Faculty of Pharmaceutical Sciences, Tokyo University of Science, Chiba 278-8510, Japan; 3b14055@alumni.tus.ac.jp (H.S.); k.mizuno_2010@stu.kanazawa-u.ac.jp (K.M.); shinaoki@rs.tus.ac.jp (S.A.); 3 Nagasaki University, Nagasaki 852-8521, Japan; deguyu0419@hotmail.co.jp (Y.D.); kzsuzuki@nagasaki-u.ac.jp (K.S.); ystanaka@nagasaki-u.ac.jp (Y.T.); ueda@nagasaki-u.ac.jp (H.U.); 4 Research Institute for Radiation Biology and Medicine, Hiroshima University, Hiroshima 734-8553, Japan; tinaba@hiroshima-u.ac.jp; 5 Department of Radiation Biology, Graduate School of Medicine, Tohoku University, Sendai 980-8575, Japan; hosoi@med.tohoku.ac.jp

**Keywords:** p53, RNA synthesis inhibitor, DNA intercalator, apoptosis

## Abstract

RNA synthesis inhibitors and protein synthesis inhibitors are useful for investigating whether biological events with unknown mechanisms require transcription or translation; however, the dependence of RNA synthesis has been difficult to verify because many RNA synthesis inhibitors cause adverse events that trigger a p53 response. In this study, we screened a library containing 9600 core compounds and obtained STK160830 that shows anti-apoptotic effects in irradiated wild-type-p53-bearing human T-cell leukemia MOLT-4 cells and murine thymocytes. In many of the p53-impaired cells and p53-knockdown cells tested, STK160830 did not show a remarkable anti-apoptotic effect, suggesting that the anti-apoptotic activity is p53-dependent. In the expression analysis of p53, p53-target gene products, and reference proteins by immunoblotting, STK160830 down-regulated the expression of many of the proteins examined, and the downregulation correlated strongly with its inhibitory effect on cell death. mRNA expression analyses by qPCR and nascent RNA capture kit revealed that STK160830 showed a decreased mRNA expression, which was similar to that induced by the RNA synthesis inhibitor actinomycin D but differed to some extent. Furthermore, unlike other RNA synthesis inhibitors such as actinomycin D, p53 accumulation by STK160830 alone was negligible, and a DNA melting-curve analysis showed very weak DNA-intercalating activity, indicating that STK160830 is a useful inhibitor for RNA synthesis without triggering p53-mediated damage responses.

## 1. Introduction

Many biological responses are controlled by the up- or down-regulation of genes and gene products through transcription, translation, and post-translational modifications. RNA synthesis inhibitors and protein synthesis inhibitors are useful for investigating whether biological phenomena with unknown mechanisms require transcription or protein synthesis. There are two modes of inhibition of mRNA synthesis by mRNA synthesis inhibitors: Direct or indirect inhibition of RNA polymerase II, both of which are known to induce a p53 response. Although the details of the mechanism of induction of the p53 response are unknown, it is thought to be a checkpoint mechanism to prevent the disruption of cellular homeostasis caused by insufficient mRNA synthesis [1].

Typical RNA synthesis inhibitors have been reported to have the ability to induce the p53 response [2,3]. Actinomycin D (Act.D) is known to cause the inhibition of RNA synthesis by intercalation between base pairs of DNA, but also induce a DNA damage response [4]. This activity has also been reported to inhibit DNA topoisomerase I and II, which, together with the inhibition of RNA synthesis, causes a p53-mediated damage response [5,6]. 5,6-Dichloro-1-b-D-ribofuranosylbenzimidazole (DRB) is a selective inhibitor of RNA polymerase II-mediated transcriptional elongation and inhibits mRNA synthesis in eukaryotic cells. Its inhibitory activity is based on inhibition of the kinase activity of P-TEFb [7,8,9], which phosphorylates the C-terminal domain of the largest subunit of RNA polymerase II, resulting in the induction of the p53 response [2]. Other RNA synthesis inhibitors, such as α-amanitin and H7, have also been reported to trigger the p53 response [2,3].

The role played by RNA synthesis inhibitors and protein synthesis inhibitors in early studies of apoptosis was important. For example, these drugs revealed that radiation-induced thymocyte apoptosis requires *de novo* RNA synthesis and protein synthesis [10,11]. After this discovery, it became clear that radiation cell death in thymocytes is p53-dependent, as thymocytes derived from p53 knockout mice were resistant to radiation-induced apoptosis [12,13]. These findings were clearly consistent with the requirement for RNA synthesis and protein synthesis, as the mechanism by which p53 executes apoptosis is a transcriptional activator. However, as mentioned earlier, Act.D, a conventional RNA synthesis inhibitor used in these reports, has DNA-damaging effects due to its own DNA-intercalating activity, which alone induces apoptosis in thymocytes and T cell-derived cell lines [14,15]. Although transactivation by p53 should be required for the execution of DNA-damage-induced p53-induced apoptosis, even in Act.D-induced apoptosis, subsequent studies have revealed that p53 itself has apoptogenic activity that allows it to translocate to mitochondria and induce apoptosis in a transcription-independent manner [3,16,17,18,19]. The fact that p53 can carry out apoptosis in the absence of transcriptional activation of p53-target genes made it difficult to verify the RNA synthesis requirement of the apoptotic cells under study. For example, we and others have reported that the human T-cell leukemia cell line MOLT-4, which is highly radiosensitive and exhibits p53-dependent radiation apoptosis [20], is not suppressed by radiation-induced apoptosis even in the presence of Act.D, concluding that the contribution of the p53 transcription-independent pathway is dominant in the irradiated cells [21,22]. However, if the induction of cell death in the presence of Act.D was due not only to radiation but also to the DNA-damaging activity of Act.D, this conclusion needed to be revised.

In this study, we screened a library containing 9600 core compounds (Drug Discovery Initiative, The University of Tokyo) [23] using some cell viability assays and obtained STK160830 that shows a high anti-apoptotic effect in irradiated MOLT-4 cells. Subsequent studies revealed that STK160830 is a novel inhibitor for mRNA synthesis. In addition, unlike Act.D, this inhibitor has characteristic activity that has very weak DNA-intercalating activity and hardly induces a p53-mediated damage response. At the same time, it became clear that the previous conclusion that *de novo* RNA synthesis is not required for radiation cell death of MOLT-4 cells needs to be revised. This finding could not have been found without the RNA synthesis inhibitory activity of STK160830, which scarcely induces a harmful p53 response. These findings indicate that STK160830 is an effective inhibitor to validate the requirement for transcription.

## 2. Materials and Methods

### 2.1. Cell Culture and Treatment

**Figure 1:**
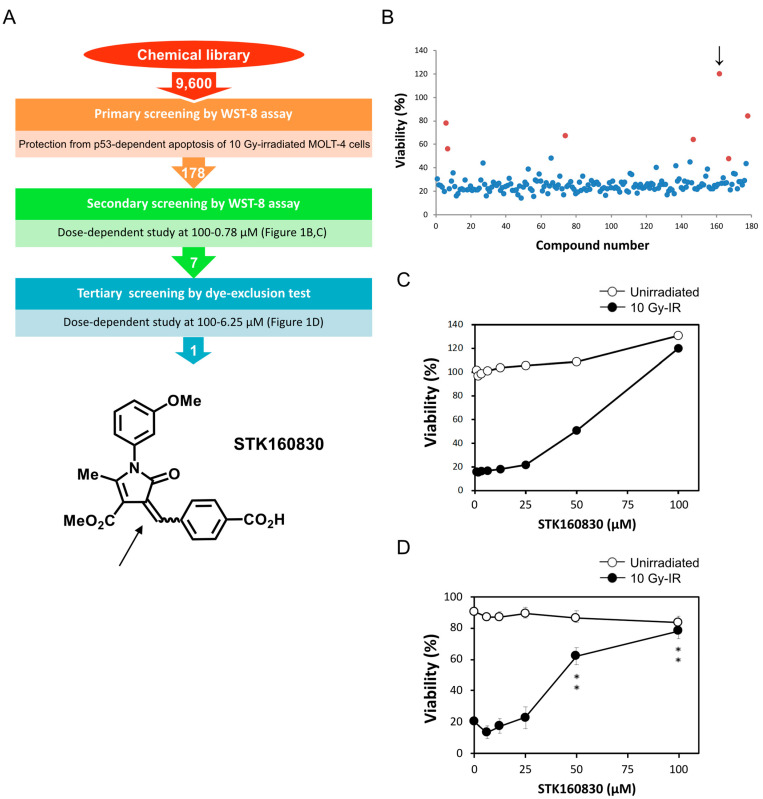
Discovery of STK160830 as a p53-dependent apoptosis inhibitor from 9600 compounds in the core library from the Drug Discovery Initiative. (**A**) Summary diagram of drug discovery screening steps, and the structure of STK160830. The double bond that gives rise to stereoisomers (*Z* and *E* forms) is indicated by an arrow. (**B**) Secondary screening by WST-8 assay selected 7 compounds that gave 2-fold higher viability than control-irradiated cells (red circles). The viability of MOLT-4 cells was measured by WST-8 assay 24 h after 10 Gy-IR. The highest viability shown by each compound in its dose-dependent test (100–0.78 µM) was dot-plotted. The arrow indicates STK160830. (**C**) Results of dose-dependent test of STK160830 in the secondary screening. Means from two independent experiments are shown. (**D**) Results of dose-dependent test (100–6.25 µM) of STK160830 in the tertiary screening. The viability was measured by dye-exclusion test 21 h after 10 Gy-IR. Means and SDs from four independent experiments are shown. **, *p* < 0.01.

The human T-cell leukemia MOLT-4 cell line bearing wild-type *TP53* [20,24,25,26,27,28] was established and gifted from Dr. Jun Minowada (Roswell Park Memorial Institute), and their derivative transformed cell lines (Nega, KD-1, and R-p53-1) were established as described in our previous reports [29,30]. KU812, KY821, CCRF-CEM, Ball-1, and U937 cells were mutated or deleted for *TP53*, respectively [24,25,31,32], and were purchased from JCRB. These cell lines were cultured in RPMI 1640 medium (Wako, Japan) supplemented with 10% fetal bovine serum (FBS; Sigma) and antibiotics (100 U/mL penicillin and 0.1 mg/mL streptomycin (Nacalai Tesque, Japan). Thymocytes from ICR female mice aged 5 weeks (SLC, Inc., Japan) were prepared as described in a previous report [33], except for the composition of the medium. The composition of the medium is RPMI 1640 with supplementation of 10% FBS, 1 mM sodium pyruvate, 2 mM L-glutamine, 50 μM β-mercaptoethanol, and antibiotics. Cells were maintained at 37 °C in a humidified atmosphere containing 5% CO_2_. Cell density was determined with a cell counter (Z1 Cell and particle counter, Beckman Coulter). Exponentially growing cell cultures (5 × 10^5^ cells/mL) in tissue culture plates or flasks (Greiner) were irradiated at room temperature with an X-ray generator (MBR-1520R-3, Hitachi, Japan) operating at 150 kV–20 mA with a filter of 0.3 mm Cu and 0.5 mm Al at a dose rate of 1.6 Gy/min, or treated with etoposide (Wako). Each compound was added to the culture medium 1 h before irradiation (IR) or the etoposide treatment. Act.D and ethidium bromide (EtBr) were purchased from Nacalai Tesque. DRB was purchased from Cayman Chemical Company. The 9600 core compounds were provided by the Drug Discovery Initiative, University of Tokyo, and primary screening was performed at the Center for Therapeutic Innovation, Graduate School of Biomedical Sciences, Nagasaki University. The experiments after the primary screening were performed at the University of Tokushima or Tokyo University of Science. STK160830 was purchased from Vitas-M Laboratory. It should be noted that STK160830 is a 1:1 mixture of *Z* and *E* forms with respect to the double bond indicated by the arrow in Figure 1A and was used as a mixture of these stereoisomers in this work, because we could not separate them due to the quick isomerization of this double bond (within 1 h) in solutions and in media at room temperature even under weak light. The protein concentrations of all the protein samples were determined using the BCA Protein Assay Reagent (Thermo Fisher Scientific, Rockford, IL, USA) and equalized.

### 2.2. Apoptosis Assay

Cell viability was determined by means of the WST-8 reduction assay (Cell counting kit-8; Dojindo, Japan), MitoTracker Red CMXRos (Molecular Probes) staining, or the erythrosin B dye-exclusion test [30,33,34]. Statistical significance was determined by one-way analysis of variance (*F*-test) followed by an individual two-tailed *t*-test or Dunnett’s multiple comparison test using Microsoft Excel for Mac 2011 with the add-in software Statcel 4 (OMS publisher Ltd., Japan) unless otherwise specified. Linear regression analyses were performed using Microsoft Excel for Mac.

### 2.3. Immunoblotting Analysis

Immunoblotting was performed essentially as described in a previous report [22]. We used the following antibodies as primary antibodies: p53 (clone DO-1, sc-126 HRP, Santa Cruz Biotechnology), p21 (clone EA10, Calbiochem), PUMA (Ab-1, Calbiochem), p53DINP1 (NB100-56627, Novus Biologicals), GAPDH (clone 6C5, GeneTex), β-Actin (clone AC-15, Sigma), caspase-3 (ab90437, abcam), or caspase-7 (clone 4G2, MBL, Japan).

### 2.4. Quantitative PCR (qPCR) Analysis

qPCR analysis was performed on a Step one plus Real-Time PCR system (Applied Biosystems) as described previously [20]. Absolute quantification was performed by comparing the Ct of the unknown samples with the standard curve obtained from purified amplicons using the PCR Clean-up kit (Macherey Nagel). Nascent RNA transcripts were captured by labeling intracellular nascent RNA with 5-ethynyl-2′-uridine (EU) using the Click-iT Nascent RNA Capture Kit (Invitrogen).

The primers used in these analyses were as follows: 

*TP53* (which encodes p53), 

(forward) 5′-AGGCCTTGGAACTCAAGGAT-3′

(reverse) 5′-CCCTTTTTGGACTTCAGGTG-3′

*ACTB* (which encodes β-Actin), 

(forward) 5′-TGGCACCCAGCACAATGAA-3′

(reverse) 5′-CTAAGTCATAGTCCGCCTAGAAGCA-3′

GAPDH, 

(forward) 5′-CCCCGGTTTCTATAAATTGAGC-3′

(reverse) 5′-CTTCCCCATGGTGTCTGAG-3′

*CDKN1A* (which encodes p21),

(forward) 5′-GGTGGCAGTAGAGGCTATGGACA-3′

(reverse) 5′-GGCTCAACGTTAGTGCCAGGA-3′

*TP53INP1* (which encodes p53DINP1), 

(forward) 5′-CTGTCTAGCTGTGCATAACTCCT-3′

(reverse) 5′-CCCCATTTCATTTTGAGCTT-3′

*BBC3* (which encodes PUMA), 

(forward) 5′-AGCCAAACGTGACCACTAGC-3′

(reverse) 5′-GCAGAGCACAGGATTCACAG-3′

### 2.5. DNA Melting-Curve Analysis

Thermal denaturation experiments of calf thymus DNA (ctDNA; Sigma) in 10 mM HEPES-NaOH buffer (pH 7.4) with I = 0.1 (NaNO_3_) were performed on a JASCO V-550 UV/vis spectrophotometer (JASCO, Japan) equipped with a thermoelectric temperature controller (±0.5 °C), a stirring unit, and a 10 mm quartz cuvette. Thermal melting curves for 50 µM ctDNA were obtained by following the absorption change at 260 nm as an effect of the raising temperature (1 °C/min). The melting temperature (*T*m) value was graphically determined from the spectral data, and the Δ*T*m value for each condition was calculated from the results in the presence and absence of additives.

## 3. Results

### 3.1. STK160830 Suppresses DNA Damage-Induced Apoptosis in A p53-Dependent Manner

In the primary screening using a core library from the Drug Discovery Initiative (The University of Tokyo), 178 compounds were selected from 9600 compounds based on their inhibitory effect on apoptosis of MOLT-4 cells 24 h after 10 Gy-IR (Figure 1A). We then performed a dose-dependent study with compound concentrations of 100–0.78 µM in the WST-8 assay and selected seven compounds that gave more than 2-fold higher viability than that of control-irradiated cells (Figure 1B).

As shown in Figure 1B,C, STK160830 gave > 100% viability to MOLT-4 cells at 100 µM, which was due to its own absorption at a 450 nm wavelength in the WST-8 assay. Therefore, in the tertiary screening, a dose-dependent test of 100–6.25 µM was performed by the dye-exclusion test, and STK160830 was selected as the compound that gave the highest viability activity in this test as well (Figure 1A). Subsequently, we tested six analogous compounds provided by the Drug Discovery Initiative, and among these seven compounds, STK160830 also showed the highest radioprotective activity (data not shown). Representative results from the dye-exclusion test are shown in Figure 1D, which shows the high radioprotective activity of STK160830 against irradiated MOLT-4 cells, increasing the viability by about 60% with low cytotoxicity.

**Figure 2:**
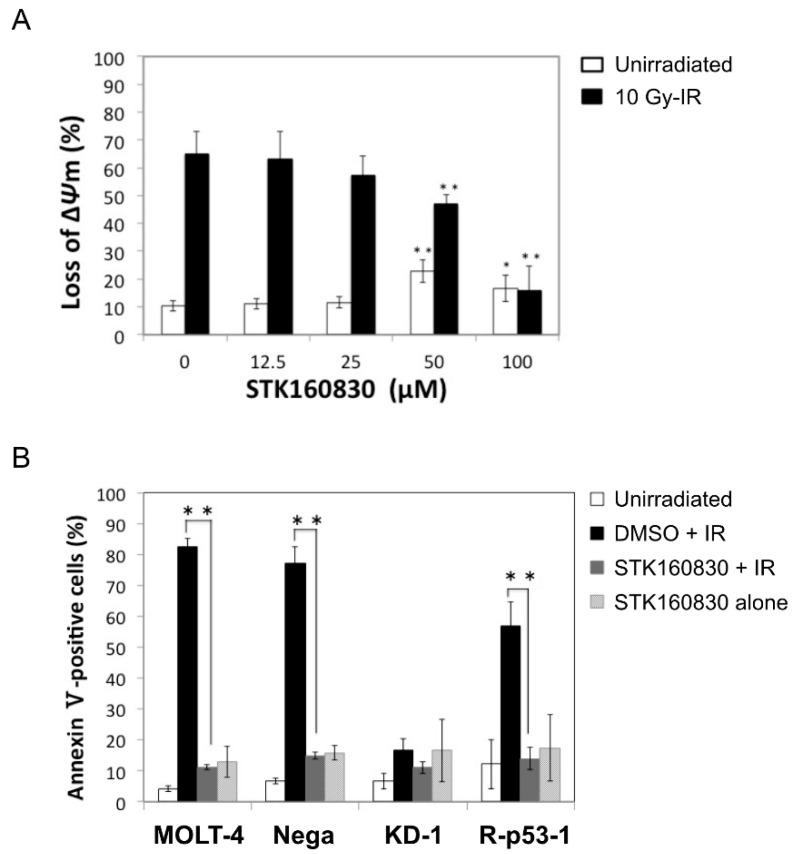
STK160830 inhibits p53-dependent mitochondrial-mediated apoptosis in irradiated MOLT-4 cells. (**A**) The percentage of MOLT-4 cells losing Δψm was measured by MitoTracker staining 15 h after 10 Gy-IR. Means and SDs from six independent experiments are shown. (**B**) The effect of 100 µM STK160830 on parental MOLT-4 cells and various MOLT-4 transfectants 16 h after 10 Gy-IR. Means and SDs from four independent experiments are shown. *, *p* < 0.05; **, *p* < 0.01.

Next, we measured whether STK160830 reduced the percentage of cells losing mitochondrial membrane potential (Δ*ψ*m) by MitoTracker staining (Figure 2A). STK160830 significantly suppressed the loss of Δ*ψ*m in irradiated MOLT-4 cells at 50 and 100 µM, indicating that it acts upstream of the mitochondria or on the mitochondria themselves.

**Figure 3:**
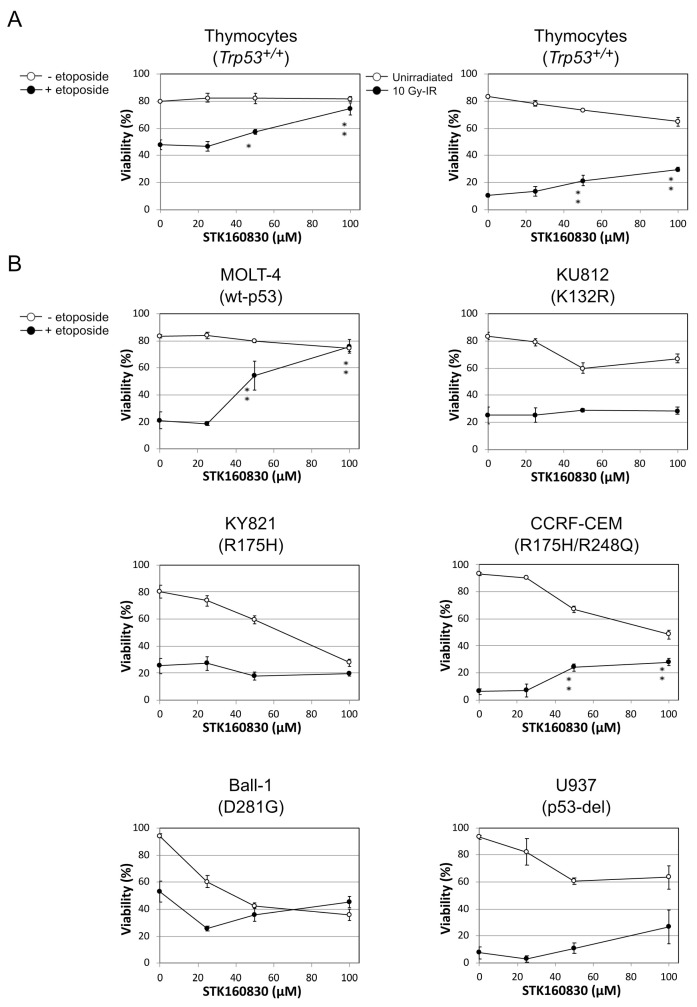
Examination of the p53 requirement for the protective effect of STK160830 on DNA damage-induced apoptosis in various cell types. (**A**) Effect of STK160830 on etoposide- or radiation-induced apoptosis in murine thymocytes. The viability was measured by dye-exclusion test 6 h after 1 µM etoposide-treatment or 8 h after 10 Gy-IR. (**B**) Effect of STK160830 on etoposide-induced cell death of MOLT-4 cells and p53-mutated KU812, KY821, CCRF-CEM, Ball-1, and U937 cells. The cell lines bearing point mutation(s) in p53 DNA-binding domain are sorted by the amino acid number. MOLT-4, KU812, KY821, CCRF-CEM, Ball-1, and U937 cells were treated with 5 µM, 50 µM, 50 µM, 2.5 µM, 5 µM, and 5µM etoposide, respectively. The viability was measured by dye-exclusion test 18 h after 10 Gy-IR. Means and SDs from three independent experiments are shown. Asterisks denote statistically significant increases in viabilities of apoptosis-stimulated cells: *, *p* < 0.05; **, *p* < 0.01.

We then assumed p53 as one of the targets of action and tested the p53 specificity of STK160830 using four cell lines: MOLT-4 (wild-type p53), Nega (mock-transfectant), KD-1 (p53-knockdown cells), and R-p53-1 (p53-revertant) (Figure 2B). STK160830 showed significant inhibition in radiation cell death of MOLT-4, Nega, and R-p53-1, in which p53 functions normally. However, no significant cell death inhibitory effect was observed in p53-knockdown cells, KD-1 (*p* = 0.15), indicating that the protective activity of this compound acts on p53. We also examined the inhibitory effect of STK160830 on DNA damage-induced apoptosis in murine thymocytes, as a typical cell type that exhibits p53-dependent apoptosis. As expected, STK160830 significantly inhibited the etoposide- or radiation-induced apoptosis of the thymocytes at 50 and 100 µM (Figure 3A). We also examined the inhibitory effect of STK160830 on etoposide-induced cell death of MOLT-4 cells and p53-mutated KU812, KY821, CCRF-CEM, Ball-1, and U937 cells by the dye-exclusion test (Figure 3B). STK160830 showed a significant inhibitory effect on etoposide-induced cell death in MOLT-4 cells, whereas it did not show a significant inhibitory effect on etoposide-induced cell death in other cells, except for CCRF-CEM cells. These results suggest that the inhibitory effect of STK160830 on DNA damage-induced apoptosis requires p53 in many cases.

### 3.2. Correlation between Changes in Intracellular Protein Expression and Viability by STK160830

**Figure 4:**
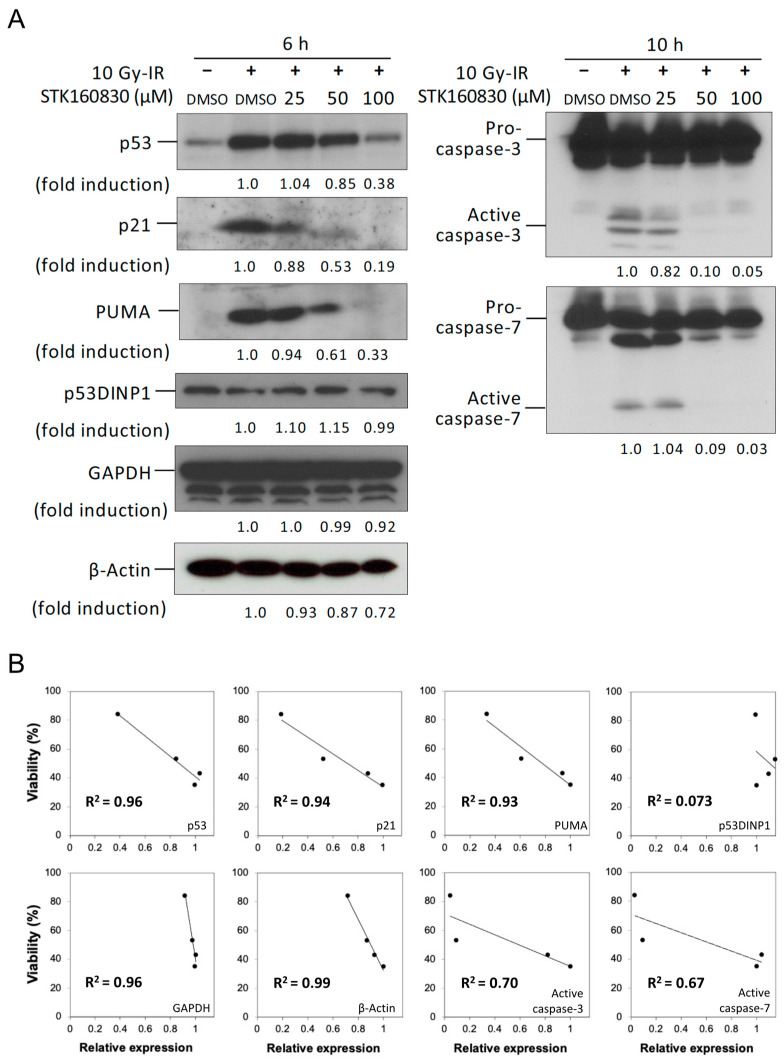
The suppressive effect of STK160830 on the MOLT-4 apoptosis was highly correlated with the suppression of various protein expression levels. (**A**) Dose–response of STK160830 on various protein expression levels. Cells were harvested 6 h after 10 Gy-IR, and the proteins were detected by immunoblotting. –, Unirradiated; +, 10 Gy-IR. (**B**) Linear regression analyses of the relationship between the irradiated MOLT-4 cell viability obtained from Figure 2A, and the relative expression levels of various proteins obtained from (**A**).

To examine the changes in protein expression in irradiated MOLT-4 cells by STK160830, immunoblotting of various proteins was performed (Figure 4A). The results showed that STK160830 suppressed protein expression of p53, p21, and PUMA, which are target gene products of p53, GAPDH, and β-Actin, which are internal standards, and activated Caspase-3 and -7 (large subunit), which are indicators of apoptotic execution. p53DINP1, a p53 target gene, was exceptionally constitutively expressed and was poorly altered by STK160830.

We then examined the correlation between the protective effect of STK160830 on viability (the percentage of cells keeping Δ*ψ*m, derived from the data in Figure 2A) and protein expression (Figure 4B). p53, p21, PUMA, GAPDH, and β-Actin showed a high correlation with a coefficient of determination (R^2^) of 0.9 or higher. The R^2^ between the induction of the large subunit of Caspase-3 and Caspase-7 and cell viability was moderate at approximately 0.7. The R^2^ for p53DINP1 was exceptionally low, at 0.073. Although the degree of repression varies among the proteins, these results suggest that the suppression of p53-dependent apoptosis by STK160830 is due to the repression of a wide range of gene expression.

Considering that the decrease in p53 by STK160830 in irradiated MOLT-4 cells might be caused by the enhancement of p53 degradation, we also examined p53 degradation via the immunoblotting of ubiquitinated high-molecular-weight p53 (Figure S1 in the supplementary). The high-molecular-weight p53 was decreased in a concentration-dependent manner by STK160830, suggesting that the p53 degradation pathway was not activated.

### 3.3. STK160830 Is A Novel mRNA Synthesis Inhibitor with A Different Spectrum of Action from Act.D

**Figure 5:**
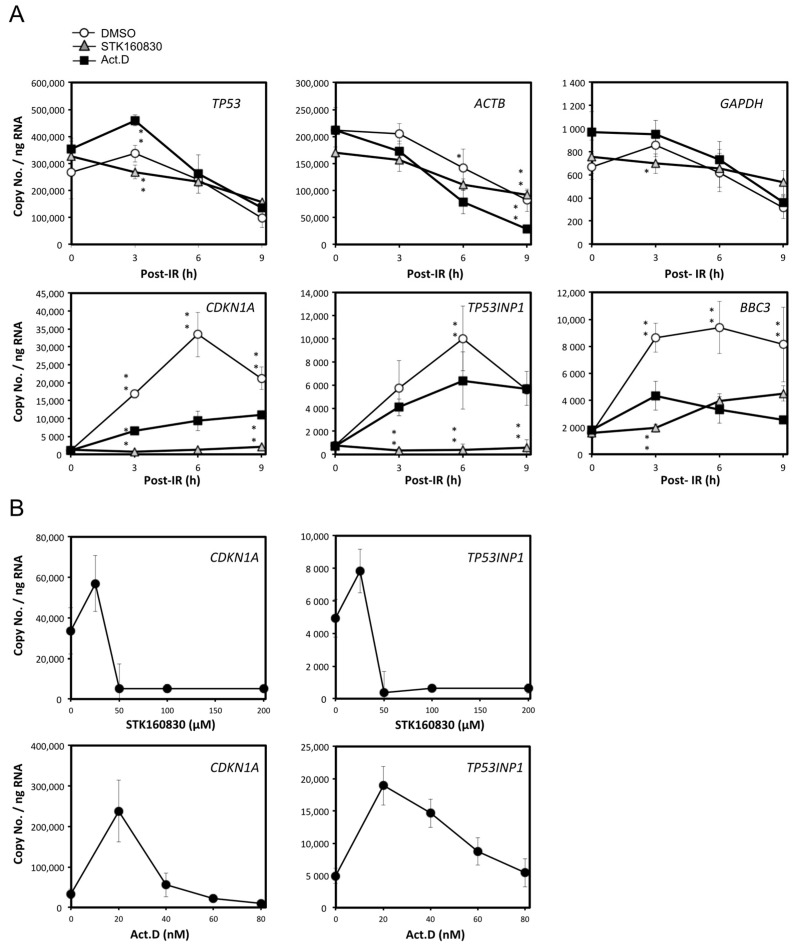
Changes in mRNA expression levels by STK160830 or Act.D in irradiated MOLT-4 cells. (**A**) STK160830 or Act.D were treated at 100 µM or 80 nM. Cells were collected at 3, 6, and 9 h after 10 Gy-IR, and subjected to qPCR absolute quantification. Means and SDs from 4–6 independent experiments are shown. Statistical significance was determined by Dunnett’s multiple comparison test using Act.D samples as controls: *, *p* < 0.05; **, *p* < 0.01. (**B**) Dose-dependent suppression of mRNA expression by STK160830 or Act.D. Cells were collected 6 h after 10 Gy-IR and subjected to qPCR absolute quantification. Means and SDs from 4–6 independent experiments are shown.

Since the amount of protein expression depends on the stability of the protein, we examined whether the suppression of gene expression by STK160830 was due to the suppression of mRNA expression. Therefore, the change in mRNA levels in irradiated MOLT-4 cells by STK160830 was examined by qPCR absolute quantification using Act.D as a control compound (Figure 5A). In the analysis of constitutively expressed *TP53*, *ACTB*, and *GAPDH*, the three treatments (vehicle DMSO, ActD, or STK160830) showed a similar decrease in mRNA expression after 10 Gy-IR. In the results for *CDKN1*A (encoding p21), *TP53INP1* (encoding p53DINP1), and *BBC3* (encoding PUMA, 3 h post-IR), STK160830 suppressed mRNA expression more strongly than Act.D. In particular, 6 h after IR, Act.D inhibited mRNA expression of *CDKN1A* and *TP53INP1* by 72% and 36%, respectively, while STK160830 highly inhibited both mRNAs by 96%.

Figure 5B shows that the treatment dose of 100 µM STK160830 and 80 nM Act.D used in Figure 5A is sufficient to inhibit the mRNA expression of *CDKN1A*, while Act.D has a slightly weaker suppressive effect on the mRNA expression of *TP53INP1*. It should be added that 80 nM Act.D, which is equivalent to 100 ng/mL Act.D, decreases the viability of non-irradiated MOLT-4 cells by 30%, and increases the viability of 10 Gy-irradiated MOLT-4 cells by only 20% [22].

**Figure 6:**
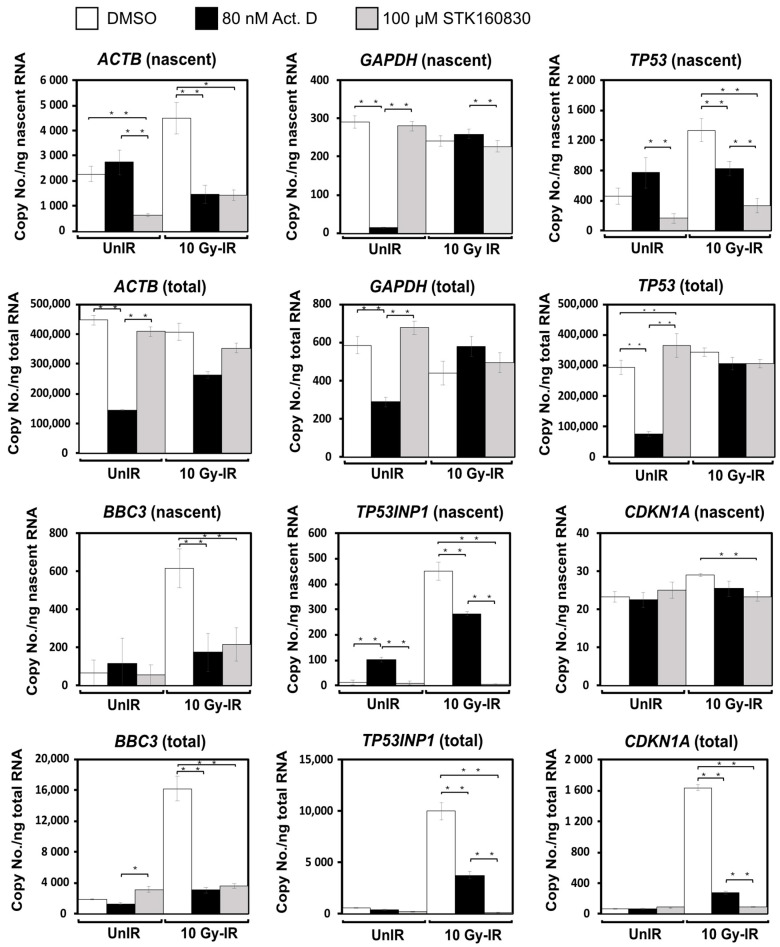
Changes in nascent and total mRNA expression by STK160830 or Act.D in irradiated MOLT-4 cells. EU was added to the culture medium immediately after 10 Gy-IR. Six hours later, total RNA was extracted from the harvested cells, and nascent RNA was isolated using the capture kit and absolutely quantified by qPCR. Means and SDs from 4–8 independent experiments are shown. Means were compared using one-way analysis of variance followed by multiple comparisons using the Tukey-Kramer method to examine significant differences: *, *p* < 0.05; **, *p* < 0.01.

We then labeled the nascent mRNA by treating the cells with EU and measured each mRNA by qPCR absolute quantification of the extracted total mRNA and the nascent mRNA obtained with the capture kit (Figure 6). In nascent mRNA synthesis of constitutively expressed genes in unirradiated cells, Act.D decreased only *GAPDH* and STK160830 decreased only *ACTB*. In nascent mRNA synthesis of inducible genes in irradiated cells, Act.D decreased *TP53*, *BBC3,* and *TP53INP1*, while STK160830 decreased *TP53*, *BBC3*, *TP53INP1*, and *CDKN1A*. For *CDKN1A*, STK160830 showed a high inhibition rate of total mRNA synthesis, whereas the inhibition effect of nascent mRNA synthesis was not remarkable compared with other genes, although it showed a significant difference. On the other hand, in unirradiated cells, the inhibitory effect of Act.D on total mRNA expression of housekeeping genes *ACTB* and *GAPDH* was higher than that of STK160830. These results suggest that STK160830 is an mRNA synthesis inhibitor with a different inhibition spectrum to Act.D. Of note, in unirradiated cells, nascent mRNA synthesis of *TP53INP1* was induced by Act.D, and that of *TP53* was also increased by Act.D treatment, albeit not statistically significant. These results suggested that Act.D activates p53.

### 3.4. STK160830 Has Negligible DNA-Intercalating Activity for Triggering A p53 Response

**Figure 7:**
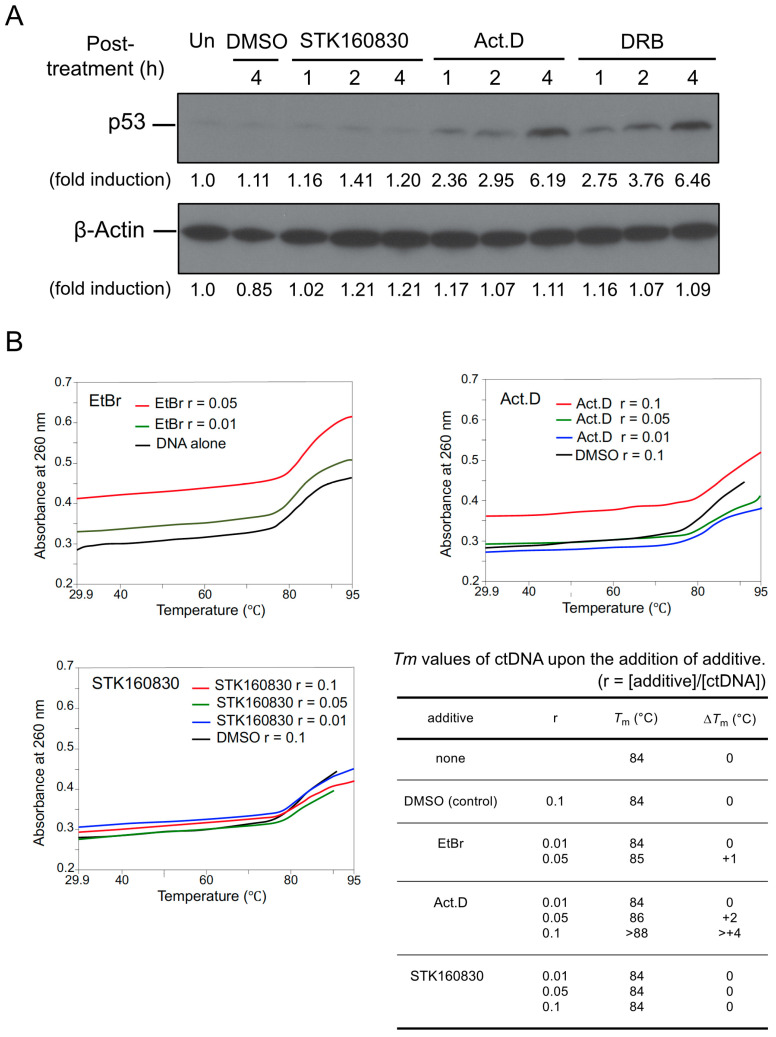
STK160830 alone does not induce p53 accumulation and has negligible DNA-intercalating activity. (**A**) Effect of STK160830, Act.D, or DRB on the induction of p53 accumulation. STK160830, Act.D, and DRB were treated at 100 µM, 80 nM, and 40 µM, respectively. Cells were collected at 1, 2, and 4 h after 10 Gy-IR, and the proteins were detected by immunoblotting. (**B**) Thermal melting curves for calf thymus DNA (ctDNA) in the presence of additives were obtained by following the absorption change at 260 nm as an effect of the raising temperature. The *Tm* value was graphically determined from the spectral data. r = [additive]/[ctDNA].

We examined the p53-inducing activity of STK160830 by immunoblotting using the conventional RNA synthesis inhibitors Act.D and DRB as controls (Figure 7A and Figure S2). Act.D and DRB induced the accumulation of p53, respectively, while STK160830 did not induce the accumulation of p53, suggesting that the inhibition of RNA synthesis is due to a mechanism different from that of the conventional inhibitors. In fact, the DNA-intercalating activity of STK160830 was also examined by measuring the *Tm* value via DNA melting curve analysis. Figure 7B shows that the well-known intercalator, ethidium bromide (EtBr) or Act.D, caused an increase in the *Tm* value compared to the control sample, whereas STK160830 did not change the melting temperature (*Tm*) value. These results indicate that STK160830 has negligible DNA-intercalating activity and a novel RNA synthesis inhibitory activity that does not trigger the p53 response.

## 4. Discussion

In this study, STK160830 was selected from the core library consisting of 9600 compounds provided from the University of Tokyo’s Drug Discovery Initiative, mainly based on its inhibitory effect on radiation-induced apoptosis of MOLT-4 cells. Of the 9600 compounds with various active compounds, the most effective compound was STK160830, which is an RNA synthesis inhibitor that has negligible DNA-intercalating activity. This indicates that *de novo* RNA synthesis is necessary for MOLT-4 cell death, but these findings are inconsistent with our and others’ conclusions that *de novo* protein synthesis, but not *de novo* RNA synthesis, is required for this cell death [21,22]. The main reason for these conclusions was that the RNA inhibitor Act.D, which was available at that time, did not block cell death. On the other hand, cycloheximide, a protein synthesis inhibitor, effectively suppressed the accumulation of p53 in irradiated MOLT-4 cells, and we speculated that the contribution of the p53 transcription-dependent pathway was probably small, because the cells had Arg72-p53, which tends to activate the transcription-independent pathway [35,36]. However, STK160830 showed the same mRNA expression inhibition at 50 µM treatment as at 100 µM (Figure 5B), and showed high radiation cell death inhibition (Figure 1D), unlike Act.D [22], even though 50 µM STK160830 showed only a slight p53 inhibition of about 15% (Figure 4A). These results suggest that the fate of the cell death is not solely dependent on the amount of p53.

In addition, in MOLT-4 cells, Act.D alone induced the p53 accumulation (Figure 7A) and had strong cytotoxicity [22], whereas STK160830 did not induce the p53 accumulation and showed almost no cytotoxicity. It is suggested that the reason Act.D showed only a small inhibitory effect on radiation-induced apoptosis in our previous study was not because the contribution of the p53 transcription-dependent pathway was small, but because the strong cytotoxicity of Act.D prevented us from observing the inhibitory effect on the MOLT-4 apoptosis. Alternatively, the inhibition of RNA synthesis by DNA-intercalation may activate the transcription-independent pathway by Arg72-p53 as a bypass pathway. In any case, the discovery of the RNA synthesis requirement for MOLT-4 apoptosis would not have been possible without the RNA synthesis inhibitory activity of STK160830, which does not induce a marked adverse response. STK160830 will be a useful RNA synthesis inhibitor, especially when investigating the transcriptional dependence of cell-death-related responses.

We present a proposed scheme for the mechanism of action of STK160830 in Figure S3. The known RNA synthesis inhibitors DRB and α-amanitin, which also lack intercalating activity, inhibit the activity of RNA polymerase II but induce a p53 response [2,3], so the target of STK160830 is presumably different from that of these conventional inhibitors. In a recent study, α-amanitin was reported to show high cytotoxic activity in cancer cells hemizygous for the deletion of one allele of *POLR2A*, the gene encoding hRBP1, one of the subunits of RNA polymerase II [37]. However, STK160830 did not show potent cytotoxicity against these cell lines (SNU283 and SW837) compared to α-amanitin (data not shown), also indicating that STK160830 has a different mechanism of RNA synthesis inhibition from that of α-amanitin.

In Figure 6, we compared the total and nascent mRNA expression levels of various genes between STK160830- and Act.D-treated cells. Although it is clear that STK160830 has a different inhibition spectrum than that of Act.D, these results are complex and difficult to interpret. As for *CDKN1A* in irradiated MOLT-4 cells, STK160830 and Act.D strongly inhibited the total mRNA synthesis. However, in the nascent mRNA expression of *CDKN1A*, STK160830 showed statistically significant inhibition of the expression, but its inhibition was slight, suggesting that it was not the inhibition of the nascent mRNA synthesis that mainly regulated the expression of total mRNA. *CDKN1A* is also a target of several miRNAs involved in T-cell acute lymphoblastic leukemia [38], suggesting that total mRNA expression of *CDKN1A* is regulated by mechanisms other than direct nascent mRNA synthesis inhibition. More interestingly, in unirradiated cells, the suppressive effect on the total mRNA expression of the housekeeping genes, *ACTB* and *GAPDH*, was observed for Act.D but not for STK160830. Since STK160830 inhibits the nascent mRNA synthesis of *ACTB* in non-irradiated cells, there may be some unknown mechanism that prevents the decrease in total mRNA. These effects of STK160830 may also contribute to its low cytotoxicity. We are currently exploring the target proteins of STK160830 using its reactive derivatives, which are expected to label them, and that should be the subject for future research.

## Data Availability

The data presented in this study are available on request from the corresponding author.

## Supplementary Materials

The following are available online at https://www.mdpi.com/article/10.3390/life11101087/s1, Supplementary Figure S1: Effect of STK160830 on the amount of high-molecular-weight p53 in irradiated MOLT-4 cells; Supplementary Figure S2: Effect of 200 μM STK160830 on the induction of p53 accumulation in irradiated MOLT-4 cells; Supplementary Figure S3: Proposed scheme of RNA synthesis inhibition and p53 accumulation by STK160830 and Act.D; Supplementary Figure S4: Uncropped blots for immunoblotting figures.
