# Supplementary material for: A Novel RNA Synthesis Inhibitor, STK160830, Has Negligible DNA-Intercalating Activity for Triggering A p53 Response, and Can Inhibit p53-Dependent Apoptosis
Source: Life (Basel). 2021 Oct 15;11(10):1087. doi: 10.3390/life11101087 (PMC8539076; doi:10.3390/life11101087)
Supplement: Supplementary file 1 [file life-11-01087-s001.zip › life-1399215-supplementary.pdf]

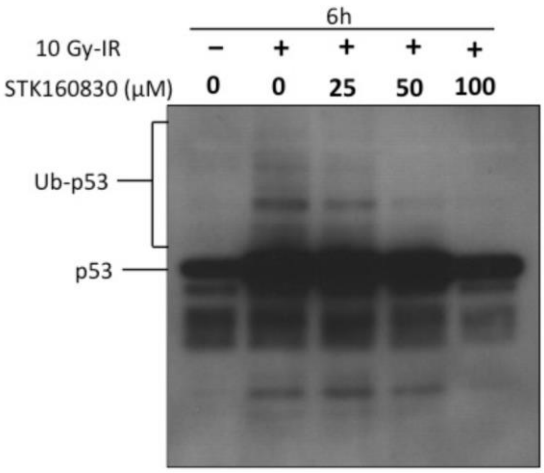

Figure S1.
Effect of STK160830 on the amount of high-molecular-weight p53 in irradiated MOLT-4 cells.
Cells were collected at 6 h after 10 Gy-IR, and the proteins were detected by immunoblotting.
The film exposure time for ECL emission was longer than usual to detect high-molecular-weight p53.

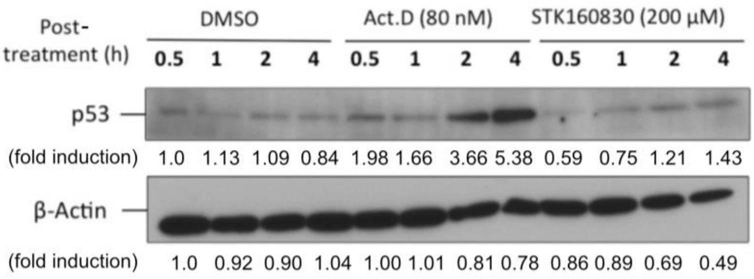

Figure S2.
Effect of 200 µM STK160830 on the induction of p53 accumulation in irradiated MOLT-4 cells.
Cells were collected at 6 h after 10 Gy-IR, and the proteins were detected by immunoblotting.
Even when the concentration of STK160830 was increased to 200 µM, the accumulation of p53 was slight.

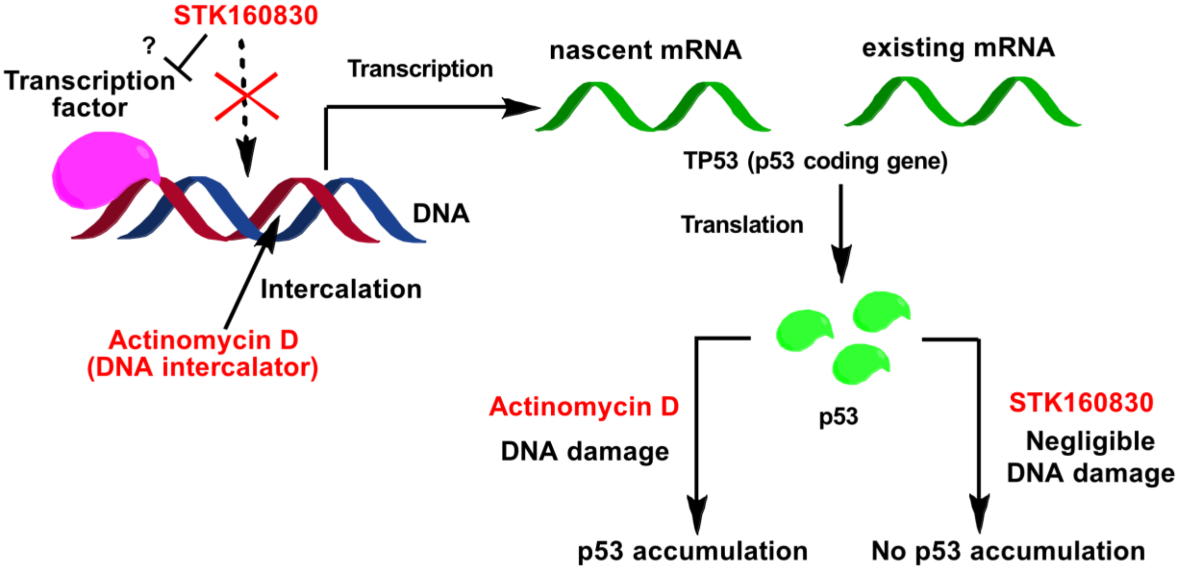

Figure S3.
Proposed scheme of RNA synthesis inhibition and p53 accumulation by STK160830 and Act.D.

Figure 4A

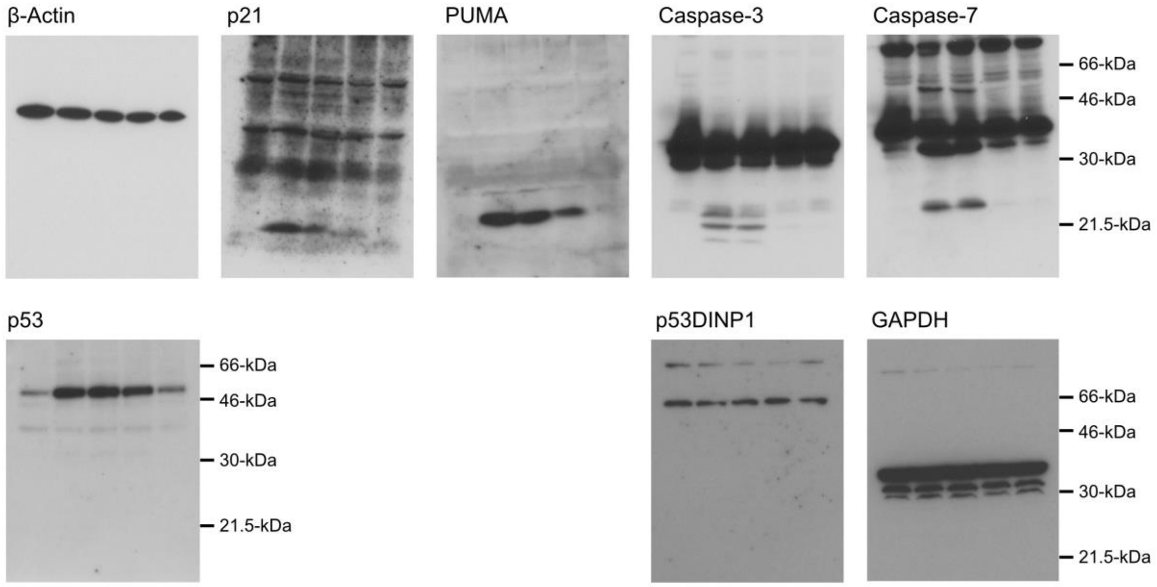

Figure 7A

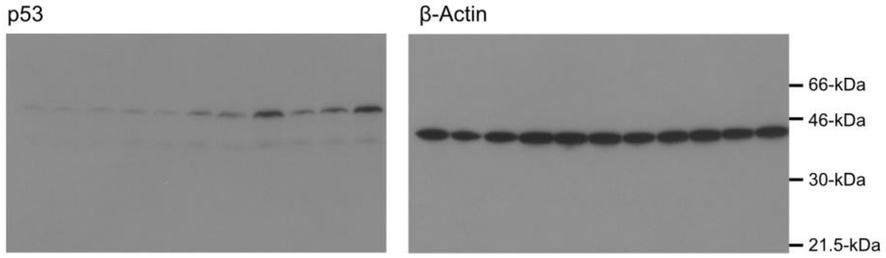

Figure S2

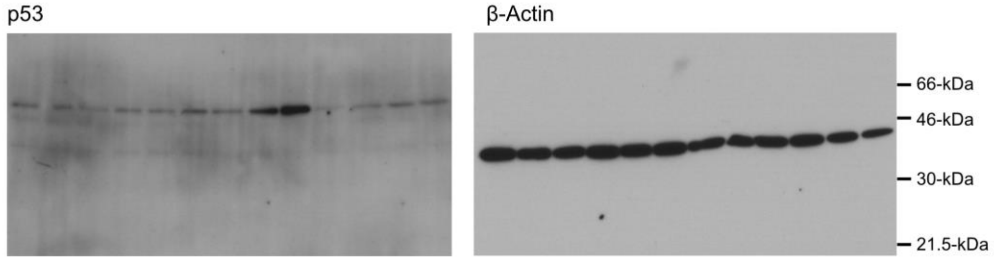

Figure S4.
Uncropped blots for immunoblotting figures.
